# Feasibility and applicability of pulmonary nodule day surgery in thoracic surgery

**DOI:** 10.3389/fsurg.2022.1013830

**Published:** 2022-09-15

**Authors:** Jiajun Han, Ruijun Zhu, Cheng Ding, Jun Zhao

**Affiliations:** Department of Thoracic Surgery, The First Affiliated Hospital of Soochow University, Suzhou, China

**Keywords:** day surgery, thoracic surgery, video-assisted thoracoscopic surgery (VATS), wedge resection, anatomic lung resection

## Abstract

**Background:**

More patients with lung diseases were identified with low-dose computed tomography (CT) popularization and increasing physical examination awareness. Day surgery was routinely conducted in many departments as a relatively mature diagnosis and treatment mode. Thus, this study aimed to assess the feasibility of day surgery in thoracic surgery for pulmonary surgery and provide guidance for selecting suitable patients.

**Methods:**

This study retrospectively analyzed the clinical data of patients with pulmonary nodule surgeries. Patients were divided into the day and routine surgery groups following chest tube removal within 48 h postoperatively and the discharge criteria. Each group was further divided into the wedge and anatomic lung resection groups. The feasibility and applicability of day surgery in thoracic surgery was evaluated by calculating the percentage of the day surgery group and comparing the clinical data of the two groups, and corresponding guidance was given for selecting suitable patients for day surgery.

**Results:**

The day surgery group accounted for 53.4% of the total number of patients in both groups. Data comparison revealed differences in age, hypertension, coronary heart disease, pulmonary function index, nodule localization, pleural adhesion, total postoperative drainage, and complications in the wedge resection and age, gender, smoking history, pulmonary function indexes, intraoperative adhesions, operative duration, total postoperative drainage volume, and complications in the anatomic lung resection (*P* < 0.05). There were no significant differences in the rates of re-hospitalization (1/172 ratio 1/150) and re-drainage (0/172 ratio 1/150) (*P* > 0.05).

**Conclusion:**

This study concluded that more than half of the pulmonary surgery can be applied to the treatment mode of day surgery, and day surgery can be applied to the screened patients. It conforms to the concept of accelerated rehabilitation and can speed up bed turnover so that more patients can receive high-level medical treatment promptly.

## Introduction

An increasing number of patients with lung diseases have been identified with the coronavirus disease-2019 outbreak and the enhanced physical examination awareness, resulting in a large number of patients who need surgery (1, 2). As a developing country, China has not yet implemented a complete hierarchical diagnosis and treatment system, and most patients have greater trust in large general hospitals; thus, the difficulty of seeking medical treatment has become increasingly prominent. Day surgery was created to alleviate the increasing shortage of beds. The International Association for Ambulatory Surgery defines day surgery as a patient's admission, surgery, and discharge within 1 working day, excluding outpatient surgery (3). China Ambulatory Surgery Alliance defines day surgery as a planned surgery that is performed with the patient discharged within 24 h, excluding outpatient surgery. The maximum duration of hospitalization is <48 h (4). Day surgery is commonly practiced in more developed countries, such as the USA and countries in Europe, as a relatively mature diagnosis and treatment mode (5). Additionally, it is routinely practiced in many departments of general hospitals in China (6), but most of them involve less traumatic operations, such as pain, urology surgery, and general surgery departments. Only large general hospitals have occasionally tried day surgery in the thoracic surgery department, which is traditionally high-risk (7, 8).

This study collected and classified the clinical data of the single medical group of thoracic surgery at the hospital of the current study following the discharge requirements of day surgery to demonstrate the feasibility, audience, and reasonable planning of day surgery in the field of thoracic surgery.

## Materials and methods

### Study design and patient characteristics

The study was conducted in accordance with the Declaration of Helsinki (as revised in 2013). This study was approved by the Ethics Committee of the First Affiliated Hospital of Soochow University (Approval No. 2022 technology 289) and informed consent was obtained from all the patients.

This study selected 399 consecutive patients in a single group of thoracic surgery, including 355 patients of surgery, from September 2021 to March 2022, at the First Affiliated Hospital of Soochow University, Jiangsu, China. Patients with mediastinal, esophageal, and bulla surgeries were excluded. Patients whose chest tubes were removed within 48 h postoperatively and met the discharge criteria were considered as day surgery mode and were defined as the day surgery group. The rest of the patients were defined as the routine surgery group. Each group was further divided into the wedge and anatomic lung resection groups following different surgical methods. The anatomic lung resection subgroup included lobectomy and segmentectomy. The clinical data were collected and compared. The feasibility of day surgery in thoracic surgery was evaluated by the proportion of patients in the day surgery group, and the clinical characteristics were compared to give corresponding guidance for selecting suitable patients for the day surgery.

### Interventions

#### Preoperative examination, education, and anesthesia assessment

All patients underwent preoperative examination and airway preparation after admission. These tests include electrocardiogram, lung function, chest CT, and abdominal ultrasound. Positron emission tomography-CT and bronchoscopy were performed for patients with solid masses and central lesions, respectively. Further echocardiography and coronary angiography were performed for older patients to evaluate cardiac function. Laboratory tests include blood routine, liver function, renal function, electrolytes, blood coagulation routine, blood type, and so on. Consultations from other departments were conducted for patients with abnormal examination results, and further examinations were performed to exclude surgical contraindications.

Both doctors and nurses informed the patient to quit smoking, do deep breathing exercises, and practice coughing on the day of admission. The nurse informed the patients about the precautions during the perioperative period by playing a multimedia video the day before the operation. An anesthesiologist conducted a preoperative visit to obtain an American Society of Anesthesiologists score. The new evidence-based fasting guidelines recommended water and solid food fasting times before anesthesia at 2 and 6 h, respectively. A certain amount of liquid was allowed to be taken before the operation.

#### Operations

Patients who met the surgical requirements and planned to undergo minimally invasive surgery underwent uniport video-assisted thoracoscopic surgery (VATS). The patient was placed on the unaffected side under general anesthesia. Double-lumen endotracheal tubes were used. The unaffected-side lung was ventilated. The utility incision was usually made at the fourth intercostal space, anterior axillary line of the upper lung; as for the lower lung, it was usually made at the fifth intercostal space, anterior axillary line. Wedge, segmentectomy, and radical lung cancer resections were performed under the principle of tumor resection following the lesion size and location and fast-freezing pathological examination. The lymph nodes were sampled in cases of adenocarcinoma *in situ* or microinvasive adenocarcinoma. Systematic lymph node dissection was performed in cases of invasive adenocarcinoma or squamous cell carcinoma. Thoracotomy was performed when the VATS was difficult.

The body temperature is monitored through a temperature-monitoring catheter during anesthesia, and the body temperature is constantly maintained through a heating blanket and a warm liquid pump. The intercostal nerve block was performed during the operation, and ∼20 ml of 1:1 mixed solution of lidocaine and ropivacaine were used, which were injected into the intercostal space and the upper and lower intercostal space where the incision was located. A #24 rigid chest tube is routinely placed in the incision, with the upper end of the chest tube at the top of the chest. Urinary catheters were not routinely indwelled during the perioperative period (9) and were only indwelled when patients had difficulty urinating postoperatively and failed to apply heat compress or change the urination position.

#### Postoperative guidance and follow-up

Patients were instructed to cough and regularly expectorate and expand the lungs after recovering from anesthesia. They could eat warm water and liquid 6 h postoperatively and are asked to get out of bed and promote lung recruitment and drainage of pleural fluid 12 h postoperatively. A chest x-ray was routinely performed 24 h postoperatively, and the chest tube was removed upon meeting the indication for extubation. All discharged patients will be distributed with a booklet of precautions and informed of the 24-h hotline for consultation after discharge.

The indications for extubation were no pneumothorax or atelectasis, no air leakage, pleural effusion is not dark in color, and a drainage volume of ≤200 ml.

The discharge criteria are as follows: chest tube has been removed, no obvious cough and other respiratory symptoms, stable vital signs, and no obvious abnormality in blood routine examination and chest x-ray.

### Assessments

Collected basic information and perioperative data included age, sex, tumor location, smoking history, underlying disease, height, weight, body mass index (BMI), pulmonary function indicators, nodule localization, pleural adhesion, thoracotomy, operative duration, operative hemorrhage, drainage volume on the first postoperative day, total postoperative drainage volume, complications and the rates of re-hospitalization and re-drainage.

Complications mainly include (1) air leakage (with postoperative air leakage lasting for >48 h), chest x-ray showing incomplete lung recruitment, water fluctuating greatly in the chest tube, and gas/pleural cavity of >30% or reintubation after extubation; (2) excessive drainage, with wedge and anatomic resection daily drainage of >150 and >300 ml, respectively; (3) pulmonary infection (with clear etiological diagnosis and imaging showing atelectasis or large exudates and with fever), a total number of white blood cells of >12 × 10^9^/L, difficult postoperative cough, and requiring bedside fiberoptic bronchoscopy for sputum suction; (4) chylothorax, with chyle test (+) and daily drainage of >500 ml.

### Statistical analysis

Statistical analyses were performed with IBM SPSS for Windows, version 26.0 (IBM Corp., Armonk, NY, USA). For quantitative data, the distribution morphology and homogeneity of variance were verified. If the data were normally distributed, Student's *t*-test was used for comparison, and results are presented as the mean ± standard deviation (mean ± SD); otherwise, median (interquartile spacing) [M (IQR)], and the Mann–Whitney *U* test was applied. Categorical data were compared using the chi-square (*χ*^2^) test or Wilcoxon rank-sum test. *P*-value <0.05 was considered significant.

## Results

This study selected 399 consecutive patients in a single group of thoracic surgery, including 355 patients of surgery, at the First Affiliated Hospital of Soochow University from September 2021 to March 2022. Finally, 322 patients who did the pulmonary nodules surgeries met the inclusion criteria. The mode of day surgery was met by 172 patients, including 105 and 67 patients who underwent wedge and anatomic lung resections, respectively. The routine surgery group consisted of 150 patients, including 55 and 95 patients who underwent wedge and anatomic lung resections, respectively.

Day surgery was satisfied with a rate of 65.6% in the wedge resection subgroup, and none were converted to thoracotomy. No significant differences were noted in sex, height, weight, BMI, smoking history, diabetes, emphysema, pulmonary surgery history, tumor lobe, operative duration, operative hemorrhage, and drainage volume on the first postoperative day (*P *> 0.05). Significant differences were found in age, hypertension, coronary heart disease, pulmonary function indexes, nodule localization, pleural adhesion, postoperative total drainage, and complications (*P* < 0.05). There were no significant differences in the rates of re-hospitalization (0/105 ratio 1/55) and re-drainage (0/105 ratio 1/55) (*P* > 0.05). The specific values are shown in Tables 1, 2.

**Table 1:** Characteristics and differences of the wedge resection subgroup.

Characteristics	Day surgery	Routine surgery	*P*-value
Age (years)	49 (23)	58 (23)	0.014
Gender			0.663
Male	38	18	–
Female	67	37	–
Height (cm)	163 (9)	160 (11)	0.667
Weight (kg)	60 (16)	60 (18)	0.814
BMI	23.00 ± 3.09	23.32 ± 3.35	0.544
Smoking	18	8	0.672
Basic diseases			
Hypertension	24	21	0.041
Diabetes	11	8	0.45
Coronary heart disease	0	4	0.023
Emphysema	4	1	0.834
Pulmonary surgery history	5	3	1
Pulmonary function indexes			
FEV1 (L)	2.74 ± 0.67	2.38 ± 0.78	0.02
FEV1%	100.1 (15.3)	96.2 (21.5)	0.035
MVV%	99.74 ± 16.79	92.13 ± 20.54	0.017
Lesion location			0.279
RUL	32	26	–
RML	15	6	–
RLL	29	12	–
LUL	9	5	–
LLL	20	6	–

**Table 2:** Perioperative-related factors in the wedge resection subgroup.

Characteristics	Day surgery	Routine surgery	*P*-value
Nodule localization	25	40	<0.001
Pleural adhesion	2	7	0.022
Thoracotomy	0	0	–
Mean operative duration (min)	58 (24)	53 (26)	0.475
Mean operative hemorrhage (ml)	10 (20)	10 (45)	0.076
Mean drainage volume on the first postoperative day (ml)	20 (50)	40 (80)	0.143
Total postoperative drainage volume (ml)	50 (70)	120 (215)	<0.001
Complications	1	26	<0.001
Air leakage	0	14	<0.001
Excessive drainage	0	0	–
Pulmonary infection	1	12	<0.001
Chylothorax	0	0	–
Re-hospitalization	0	1	0.344
Re-drainage	0	1	0.344

In the anatomic lung resection subgroup, day surgery was satisfied with a rate of 41.4%. No significant differences were noted in height, weight, BMI, basic diseases, pulmonary surgery history, tumor lobe, nodule localization, thoracotomy, operative hemorrhage and drainage volume on the first postoperative day (*P* > 0.05). Significant differences were found in age, sex, smoking history, pulmonary function indexes, pleural adhesion, operative duration, postoperative total drainage, and complications (*P* < 0.05). There were no significant differences in the rates of re-hospitalization (1/67 ratio 0/95) (*P* > 0.05) and re-drainage (0/67 ratio 0/95). The specific values are shown in Tables 3, 4.

**Table 3:** Characteristics and differences of the anatomic lung resection subgroup.

Characteristics	Day surgery	Routine surgery	*P*-value
Age (years)	55 (22)	64 (16)	<0.001
Gender			0.048
Male	22	46	–
Female	45	49	–
Height (cm)	162 (9)	165 (12)	0.57
Weight (kg)	60 (16)	61 (15)	0.856
BMI	23.67 ± 3.51	23.38 ± 3.12	0.577
Smoking	8	24	0.036
Basic diseases			
Hypertension	19	34	0.321
Diabetes	3	9	0.373
Coronary heart disease	2	2	1
Emphysema	5	8	0.825
Pulmonary surgery history	2	4	1
Pulmonary function indexes			
FEV1 (L)	2.51 (0.77)	2.20 (0.83)	0.016
FEV1%	99.23 ± 16.02	93.46 ± 19.49	0.048
MVV%	97.83 ± 16.83	87.38 ± 22.07	0.001
Lesion location			0.134
RUL	22	27	–
RML	12	10	–
RLL	21	27	–
LUL	5	6	–
LLL	7	25	–

**Table 4:** Perioperative related factors in the anatomic lung resection subgroup.

Characteristics	Day surgery	Routine surgery	*P*-value
Nodule localization	19	21	0.456
Pleural adhesion	0	10	0.016
Thoracotomy	0	5	0.148
Mean operative duration (min)	95 (36)	121 (50)	0.02
Mean operative hemorrhage (ml)	20 (50)	20 (140)	0.216
Mean drainage volume on the first postoperative day (ml)	70 (90)	80 (200)	0.536
Total postoperative drainage volume (ml)	120 (150)	530 (500)	<0.001
Complications	1	74	<0.001
Air leakage	0	43	<0.001
Excessive drainage	0	10	0.004
Pulmonary infection	1	20	<0.001
Chylothorax	0	1	1
Re-hospitalization	1	0	0.414
Re-drainage	0	0	–

## Discussion

With the rising day surgery, thoracic surgery followed its pace and is conducted in day surgery for mediastinal biopsy under mediastinoscopy (10). However, the treatment mode of day surgery is not widely accepted by doctors or patients, contrary to the traditional concept of greater trauma in thoracic surgery, and is not widely conducted in major hospitals. The applicable diseases of day surgery in thoracic surgery should be expanded, benefiting from the advanced surgical technology and developed instruments and equipment, especially the popularity of uniport thoracoscopic surgery in recent years. This study showed that more than half of patients with lung disease are suitable for the day surgery treatment mode. Formulating a series of diagnoses and treatment planning and screening out suitable patients is necessary to successfully conduct the day surgery treatment mode.

The key to the promotion of day surgery is surgical procedure optimization and a good postoperative experience for patients. Pain is the most common postoperative complaint, which is a barrier to day surgery in thoracic surgery (11). It is closely related to wound length and drainage tube placement. Uniport thoracoscopic surgery has been adopted in most operations in this medical group due to surgical instrument innovation and thoracoscopic technology advancement. Uniport thoracoscopic surgery requires an incision of ∼3 cm, which greatly reduces postoperative pain (12). Only 5 (0.02%) of the patients were converted to thoracotomy. Previous studies reported that intercostal nerve block was routinely performed to reduce postoperative pain, which can achieve an analgesic time of 24 h during the operation (13). One #24 rigid chest tube was placed in the incision after surgery for postoperative exhaust and drainage. The chest tube does not touch the diaphragm and causes breathing pain due to the relatively high position of the incision. The time of extubation was determined by the chest x-ray within 24 h postoperatively. Most of the patients were within the effective analgesic time of intercostal nerve block. Early extubation not only relieves postoperative pain but also facilitates early movement (14). Many of the enhanced recovery after surgery protocols include early chest tube removal as a key step (15), although some studies suggest that discharge with a tube does not increase the risk of readmission (16). In terms of patient acceptance and comfort, discharge after extubation was significantly better than discharge with the tube; thus, all patients in this study were discharged after extubation. A urinary catheter was not routinely placed and the monitoring equipment was removed as soon as possible in addition to placing a chest tube in the surgical incision, all of which are beneficial to reducing the postoperative discomfort of patients.

The main component of day surgery, preoperative examination and education, evaluation and implementation of anesthesia, perioperative care, and postoperative follow-up also need to be in place in addition to surgery. (1) All preoperative examinations can be done before hospitalization. A reasonable appointment system can automatically match the order and time of examinations for medical resources to be more effective. It will be a win–win for both doctors and patients as long as this part of the expenses is reimbursed in the same proportion as hospitalization. For preoperative education, patients can certainly obtain videos and text materials when making an appointment for day surgery. In this way, smoking patients can quit smoking before surgery, all patients can exercise their coughing and deep breathing skills at home, and they will have a reasonable diet and exercise postoperatively. (2) This study routinely performed preoperative anesthesia assessment by an anesthesiologist in the ward. However, many general hospitals, including the hospital of the current study, have opened anesthesia outpatient clinics, which can be used to assess the risk of anesthesia, which were tried by patients with good results. Concurrently, the short preoperative fasting time and the constant intraoperative body temperature are beneficial to the postoperative recovery of patients (17). (3) Drainage position treatment, chest tube, and drainage bottle is the difficult point of postoperative care. However, all patients had their chest tubes removed when they were discharged from the hospital, which solved this difficulty. Moreover, patients are encouraged to eat and exercise early to promote physical recovery. (4) Discharge is not the end of rehabilitation. Patients recover at home or in community hospitals after they were discharged, and more patients choose the former. The nursing department will distribute the postoperative booklet to obtain the necessary guidance, and the department of the current study has set up a 24-h consultation hotline to facilitate the consultation of the discharged patients. Various emergencies can be resolved timely and effectively. This greatly relieves the anxiety of patients due to early hospital discharge.

Obtaining understanding and recognition from the patients and their families is necessary to select the appropriate patients. This is inseparable from the communication between doctors and nurses and the display of successful cases. Meeting these indicators is more important. The study revealed significant differences in age, sex, smoking history, pulmonary function indexes, pleural adhesion, operative duration, and postoperative total drainage in the wedge resection subgroup. Significant differences were found in age, hypertension, coronary heart disease, pulmonary function indexes, nodule localization, pleural adhesion, and postoperative total drainage in the anatomic lung resection subgroup. Some of the differences are found in other literature (18–21). Additionally, some literature suggests that it is related to surgical lobes and minimally invasive surgery (18, 19). This study revealed no statistical correlation between the lung lobe where the lesions were located, which may be related to the small sample size and disease incidence. However, all patients who were converted to thoracotomy resulted in delayed discharge; thus, further sample size expansion may lead to differences. Nodule localization is used when the lesion is deep, and the lesion cannot be accurately found during the operation. More lung tissue is often required to be removed because of the deeper location. The difference in wedge resection may be related to the enlarged scope of surgical resection. Some patients will have obvious pain memory, which causes certain difficulties for early discharge because the nodule localization occurs before general anesthesia.

The study found that the complications during hospitalization mainly included air leakage, excessive drainage and pulmonary infection, and there were differences between the groups, which was also the main reason for the delayed extubation and discharge of patients in the routine surgery group. Because of the broad definition of postoperative complications in this study, the incidence was high. Although we encouraged all patients to exercise and cough after operation, due to individual differences, some patients did not do well, resulting in slower lung recruitment. When the water in the chest tube fluctuated greatly, extubation might be delayed. Although some studies had pointed out that the daily drainage was more than 300 ml (22), or even 400 ml (23), it would not increase the risk of re-drainage after surgery. A daily drainage volume of ≤200 ml for extubation was set in our center. Elevated white blood cell counts are one of the bases for judging postoperative infection. Although there were also many cases of increased reactivity, discharge was only allowed when the patient's number of white blood cells were normal or close to normal after observation or anti-inflammatory.

Unplanned re-hospitalization rates are important indicators for assessing safety, and it has suggested that an acceptable re-hospitalization rate should be between 1% and 2% (24). Benefiting from stricter extubation indications and active treatment, only two patients were readmitted to the hospital. One patient in the day surgery group was readmitted for bronchial asthma which may be related to the surgery. One patient in the routine surgery group was readmitted for reintubation due to pleural effusion. Due to the 24-h consultation hotline, the patients contacted the hospital in time, and were treated immediately. There was no significant difference in the rates of re-hospitalization and re-drainage between the two groups, and it could be seen that the mode of day surgery was safe.

Therefore, caution should be exercised when the following conditions exist in planning day surgery: (1) elderly patients; (2) patients with poor pulmonary function; (3) preoperative CT suggests pleural adhesions in patients with severe adhesions during surgery; (4) patients who plan to undergo wedge resection and have underlying diseases, especially hypertension and coronary heart disease; (5) patients who are going to undergo wedge resection and need nodule localization before surgery; (6) male patients undergoing anatomic lung resection, especially with a history of smoking; and (7) patients whose operative duration is longer than expected.

This single-center retrospective study had a relatively small sample size, which may result in some bias. It only simulates the diagnosis and treatment process of day surgery in a general ward. Many difficulties need to be overcome in actual practice. All surgeries were performed by the same team of surgeons. Long-term survival outcomes need to be monitored and investigated. Therefore, a larger sample size needs to be considered in the future, and prospective comparative studies in other teams or hospitals are warranted.

## Conclusions

In summary, the treatment mode of day surgery applies to a large number of patients who underwent pulmonary surgery. It can accelerate bed turnover in line with the concept of accelerated rehabilitation so that more patients obtain a higher level of timely medical treatment.

## Data Availability

The raw data supporting the conclusions of this article will be made available by the authors, without undue reservation.
